# Affiliation, Aggression, and Selectivity of Peer Relationships in Meadow and Prairie Voles

**DOI:** 10.3389/fnbeh.2019.00052

**Published:** 2019-03-19

**Authors:** Nicole S. Lee, Nastacia L. Goodwin, Katherine E. Freitas, Annaliese K. Beery

**Affiliations:** ^1^Neuroscience and Behavior Program, University of Massachusetts, Amherst, MA, United States; ^2^Department of Psychology, Smith College, Northampton, MA, United States; ^3^Neuroscience Program, Smith College, Northampton, MA, United States

**Keywords:** meadow vole, prairie vole, social behavior, affiliation, aggression, partner preference

## Abstract

Relationships between adult peers are central to the structure of social groups. In some species, selective preferences for specific peers provide a foundation for consistent group composition. These preferences may be shaped by affiliation toward familiar individuals, and/or by aversion to unfamiliar individuals. We compared peer interactions in two vole species that form selective preferences for familiar same-sex individuals but differ in mating system. Prairie voles (*Microtus ochrogaster*) form pair bonds with mates and may reside in family groups. Meadow voles (*Microtus pennsylvanicus*) are promiscuous breeders that form communal winter groups in the wild, and exhibit greater social behavior in short day (SD) lengths in the laboratory. We characterized affiliative, anxiety-like, and aggressive interactions with familiar and novel same-sex conspecifics in meadow and prairie voles housed in summer- or winter-like photoperiods. Species differences in affective behaviors were pronounced, with prairie voles exhibiting more aggressive behavior and less anxiety-like behavior relative to meadow voles. Meadow voles housed in short (vs. long) day lengths were more affiliative and more interactive with strangers; prosocial behavior was also facilitated by a history of social housing. Prairie voles exhibited partner preferences regardless of sex or day length, indicating that selective peer preferences are the norm in prairie voles. Prairie vole females formed preferences for new same-sex social partners following re-pairing; males were often aggressive upon re-pairing. These data suggest that preferences for familiar peers in prairie voles are maintained in part by aggression toward unfamiliar individuals, as in mate partnerships. In contrast, social tolerance is an important feature of meadow vole peer affiliation, demonstrated by low aggression toward unfamiliar conspecifics, and consistent with field data on winter tolerance.

## Introduction

Relationships between non-mate group members are the foundation of social groups for many mammals, from same-sex bachelor herds and multi-female breeding groups to mixed-sex winter huddling groups in reproductively quiescent voles (Clutton-Brock, 2016; Smith et al., 2017; Lee and Beery, 2019). Numerous studies have provided insight into pathways involved in reproductive relationships—such as between a mother and her offspring, or between socially monogamous mates—but these pathways may or may not generalize to non-reproductive relationships between adult peers. Peer relationships themselves are not all the same. For example, they may be selective or non-selective (Lee, 1994), transient or enduring (Lidicker and Patton, 1987), and motivated or not motivated (Goodwin et al., 2018). We sought to characterize and compare the contributions of affiliation, anxiety, and aggression to selective relationships between peers, using monogamous and promiscuous vole species that both form partner preferences for a familiar same-sex peer. These studies provide a foundation for the comparison of reproductive and non-reproductive social preferences, and inform our understanding of factors shaping peer relationships.

The meadow vole (*Microtus pennsylvanicus*) is a promiscuous, uniparental vole species (Getz, 1972; Boonstra et al., 1993) that has been studied for its seasonal peer relationships (reviewed in Beery, 2019). In the summer reproductive season, meadow voles are intolerant of other individuals: females defend distinct territories, while males roam across multiple female territories. In the winter non-breeding season, meadow voles form social groups. They exhibit shared home ranges, nest with conspecifics, and are highly tolerant of one another (Madison, 1980; McShea and Madison, 1984; Ferkin and Seamon, 1987; Madison and McShea, 1987). In laboratory settings, meadow voles form selective, long-lasting preferences for known peers, demonstrated in the partner preference test (PPT)—in which animals can choose to spend time with a familiar or unfamiliar conspecific (Parker and Lee, 2003; Beery et al., 2008; Ondrasek et al., 2015). Laboratory manipulation of photoperiod from summer-like long days (LDs) to winter-like short days (SDs) drives variation in multiple social behaviors in parallel to seasonal variations in the field (Ferkin and Seamon, 1987; Ferkin and Gorman, 1992; Beery et al., 2008; Ondrasek et al., 2015).

The closely related but socially monogamous prairie vole (*Microtus ochrogaster*) has been an important study organism for research on parental behavior and pair bonding between mates (Carter, 2017; Gobrogge et al., 2017; Walum and Young, 2018). Unlike meadow voles, prairie voles in the wild form selective, long-lasting mate relationships and provide bi-parental care. From late autumn and through winter, prairie voles also display an increase in communal groups (extended family groups with unrelated adults) due to decreased dispersion by philopatric young, despite territoriality and largely exclusive male-female pairs at other times (Getz et al., 1993; Getz and Carter, 1996). Therefore, adult-adult same-sex cohabitation occurs in both species of voles under natural conditions.

In the two studies to date that assessed peer (same-sex) partner preferences in prairie voles, LD-housed prairie voles—like meadow voles—displayed partner preferences for a same-sex partner after 24 h of cohabitation (DeVries et al., 1997; Beery et al., 2018). Other studies of peer affiliation in female prairie voles have provided evidence that these peer relationships constitute social attachments by examining the effects of separation on anxiety- and depressive-like behaviors, as well as social buffering. Socially isolated female prairie voles displayed increased anxiety- and depressive-like behaviors compared to voles housed with same-sex siblings (Grippo et al., 2008). Isolation also caused neuroendocrine disturbances, changes in adult neurogenesis, and autonomic regulation of the heart (Grippo et al., 2007a,b). Furthermore, adult neurogenesis and autonomic regulation of the heart differed between socially isolated female prairie voles and voles housed with an unfamiliar female (Fowler et al., 2002; Grippo et al., 2007c). Social interactions may also buffer the experience of exogenous stressors, with male and female prairie voles displaying increased grooming of same-sex cage-mates that had undergone a stressor (Burkett et al., 2016).

The selectivity of peer relationships demonstrated in PPTs may arise from prosocial factors favoring a familiar partner, antisocial factors disfavoring unfamiliar individuals, or both. Aggression toward non-mate conspecifics is an important factor in the maintenance of pair bonds between mated prairie voles (Resendez et al., 2016), and may also play a role in shaping the specificity of affiliative peer relationships. For instance, female prairie voles become more aggressive toward other females after 8 days of cohabitation with a male, as well as during pregnancy (Bowler et al., 2002). Trios consisting of two females and a male exhibit higher female-female huddling when the two females are siblings, but higher aggression when the two females are unrelated (Firestone et al., 1991). Furthermore, both male and female prairie voles become more aggressive toward same-sex strangers after mating (Young et al., 2011). In males, upregulation of dopamine D1-like receptors in the nucleus accumbens corresponds with pair bond maintenance—specifically, with aggression toward unfamiliar females (Aragona et al., 2006). Blocking these receptors also reduced the aggressive behavior. Thus, both affiliative and aggressive interactions mediate important aspects of the social organization of prairie voles, and may shape peer interactions.

We compared characteristics of peer social relationships within and across monogamous and promiscuous vole species, focusing on affiliative, aggressive, and anxiety-like behaviors. Prior studies have demonstrated the presence of same-sex partner preferences in meadow and prairie voles, existence of sex and day length differences in meadow vole peer partner preferences, and sex differences in prairie vole peer partner preferences in LDs (DeVries et al., 1997; Beery et al., 2008, 2009; Ondrasek et al., 2015). We asked: (1) whether there are species differences (prairie vs. meadow) in affiliation and aggression toward an unfamiliar same-sex peer, or in anxiety-related behaviors (study 1a); (2) whether meadow voles exhibit photoperiodic changes in aggressive behavior related to changing seasonal social tolerance (study 1b); and (3) whether partner preferences in prairie voles are modulated by day length, as in meadow voles, and whether they can form preferences for new peer partners following separation and re-pairing (study 2). For several of these questions we had no basis for predicting a specific outcome, but based on prior work in male prairie and meadow voles, we hypothesized that female prairie voles would be less anxious than meadow voles (Stowe et al., 2005). We also expected that there would be more intraspecific aggression in meadow voles, based on data from an interspecific comparison of voles trapped in Illinois and Michigan (Getz, 1972). Finally, we predicted that SD-housed meadow voles would likely be less aggressive than LD-housed meadow voles, consistent with increased sociality in short photoperiods and winter field conditions. We relate differences in prairie and meadow vole aggression and anxiety to differences in peer affiliation. This study lays the behavioral foundations necessary for comparative work on mechanisms underlying social behaviors in meadow and prairie voles.

## Materials and Methods

### Animal Subjects

Prairie and meadow voles were bred locally at Smith College as described in Goodwin et al. (2018). Animals were group weaned at 19 ± 1 days (meadow voles) or 21 ± 1 days (prairie voles), then separated to pair-housing with either a same-sex sibling or an age-matched same-sex non-sibling (about half to each type of pairing) within 1 week. One meadow vole group was weaned into solo-housing. Voles were maintained on a LD light cycle (14 h light; 03:00–17:00 EST) or transferred to a SD light cycle (10 h light; 07:00–17:00 EST) at weaning. Voles were housed in clear plastic cages (45 × 25 × 15 cm) with aspen bedding (Envigo TekLab), nesting material (Lab Supply Enviro-dri and a nestlet), and a PVC hiding tube. Rooms were maintained at approximately 20°C, and food (Labdiet Mouse Chow 5015 for meadow voles, and 5015 mixed with Rabbit Chow 5326 for prairie voles) and water were available *ad libitum* with every-other-day supplementation of apple or carrot.

Voles were 80 ± 7 days of age at the start of testing. This study was carried out in accordance with the recommendations in the Guide for the Care and Use of Laboratory Animals published by the National Research Council. The protocol was approved by the Institutional Animal Care and Use Committee at Smith College.

### Experimental Design

#### Study 1a: Species Differences in Novel Social Interactions, Aggression, and Anxiety

Focal voles were tested for affiliative, aggressive, and anxiety-like behaviors. Subjects were pair-housed SD meadow vole females (*n* = 19), and pair-housed SD prairie vole females (*n* = 17). At 80 ± 7 days of age, focal voles were tested for aggressive and affiliative behaviors in a social interaction test in a neutral arena with an unrelated, same-sex, novel stranger. One week later, focal voles underwent open field tests and light-dark box tests (OFTs and LDB, respectively) on consecutive days.

#### Study 1b: Effects of Day Length and Social History in Meadow Voles

Pair-housed SD meadow vole females from Study 1a were also compared to pair-housed LD meadow vole females (*n* = 19), and solo-housed SD meadow vole females (*n* = 16), in the social interaction test. The solo-housed LD meadow vole female group was solo-housed from weaning. At 80 ± 7 days of age, focal voles were tested for aggressive and affiliative behaviors in a social interaction test in a neutral arena with an unrelated, same-sex, novel stranger.

#### Study 2: Formation and Reformation of Peer Partner Preferences in Prairie Voles

A separate cohort of voles was tested for the strength of preferences for familiar same-sex social partners after cohabitation with a partner from weaning (PPT 1). A subset was re-paired with a new partner in adulthood and tested after 24 h (PPT 2).

PPT 1: subjects were pair-housed SD prairie vole males (*n* = 13), SD prairie vole females (*n* = 11), and LD prairie vole females (*n* = 14). At 80 ± 7 days of age, focal voles were tested for partner preference for their cage-mate since weaning, as described below. The ability to form new same-sex partner preferences in adulthood was assessed in PPT 2: eight subjects from each group were separated from their partners 24 h after PPT 1. Voles were housed alone for 8 days, after which they were cohoused with novel, unrelated, same-sex partners. Focal voles underwent partner preference testing after 24 h of cohabitation with these new partners. The male sub-group was stopped early because of a high rate of aggression upon re-pairing, and is not included in the formal analysis.

### Behavioral Testing

#### Partner Preference Test

Peer partner preference testing was conducted in a rectangular plastic apparatus consisting of three equal-sized compartments arranged linearly (75 × 20 × 30 cm), as previously described (Anacker et al., 2016a,b; Beery et al., 2018). The cage-mate of the focal vole (the partner) was tethered at one end of the apparatus, and an age-matched, unrelated, same-sex novel vole (the stranger) was tethered at the other end. Strangers were pair-housed from weaning, and were used no more than three times over the course of Study 2. The focal vole was placed in the center chamber and allowed to move freely for the duration of the 180-min test. Tests were video recorded, and trained observers used custom software (Intervole Timer1.6.pl, AKB) to quantify the amount of time focal voles spent huddling (side-by-side or one on top of the other), duration in each chamber, and number of times the focal vole crossed between chambers. Partner preference in a group was defined as significantly more time huddling with the partner than the stranger; partner preferences in individuals were defined as twice as much huddling with the partner as with the stranger, as in prior studies (Insel et al., 1995; Beery et al., 2009). Scorers were blind to subject groups and position of the partner/stranger.

#### Aggression/Social Interaction Test

Interactions with an unfamiliar vole were assessed in a neutral arena. For voles, prior research has suggested that aggression is as high in a neutral arena as in home-cage tests (i.e., resident-intruder tests; Harper and Batzli, 1997). The focal vole was placed in a new cage and allowed to acclimate for 10 min. An unrelated, unfamiliar stranger of matched species, sex, day length, and housing condition was marked for identification, then introduced into the cage. The test was recorded for 10 min, or was terminated early after three significant bouts of aggression. All voles were assessed for latency to attack; a latency of 10 min (the full test duration) was recorded for voles that never attacked. Tests were scored by an observer blind to subject condition, using JWatcher 0.9 (University of California, Los Angeles and Macquarie University, Sydney) to measure the frequency and duration of behaviors, and latency to behaviors. Aggressive behaviors quantified included lateral attack/threat, upright (boxing), chasing, and clinch (as in Koolhaas et al., 2013). Clinch refers to a behavior in which the voles scuffle but are not upright, and one vole is on the bottom belly up. Social and investigative behaviors included sniffing and social exploration, grooming, and huddling. Test scoring focused on the behavior of the focal vole.

Behavior in the meadow vole groups was quantified across the full 10-min test interval. Most prairie vole pairs reached the criterion for early separation, so for species comparisons of detailed behavior (study 1a), the first 1 min 51 s of testing were used—the longest interval that could be compared across all subjects.

#### Open Field/Light-Dark Box Tests

Animals were placed at the edge of the OFT and the dark portions of the LDB test, and filmed for 5 min from above. The OFT consists of a circular open arena (42 cm diameter). Behaviors assessed included time in the center of the OFT arena (≥7 cm from the edge), distance traveled, and the number of fecal boli deposited. The LDB (49 × 20.5 × 19.5 cm) consists of a black Plexiglas box attached to a clear lidless Plexiglas box. Behaviors assessed included time in the light portion of the LDB, and latency to emerge into the light.

### Data Analysis

Differences between species were assessed with Student’s *t*-test (study 1a). Differences between multiple groups were assessed by one-way ANOVA (study 1b and 2). Significant ANOVAs were followed by two pair-wise comparisons between groups differing in one variable (study 1b: day length or housing; study 2: species or day length) using Fisher’s PLSD for normally distributed data, or Wilcoxon rank sum tests for pair-wise comparisons on data that violated normality, assessed with Shapiro-Wilk *W* tests. Wilcoxon matched-pairs signed rank tests were used for within-group comparisons of partner vs. stranger huddling.

Statistical analyses were performed in JMP 8.0 (SAS, Inc.) or GraphPad Prism 7. All tests were two-tailed. Results were deemed significant at *p* < 0.05.

## Results

### Study 1a: Species Differences

#### Species Differences in Stranger-Directed Interactions

**Figure 1:**
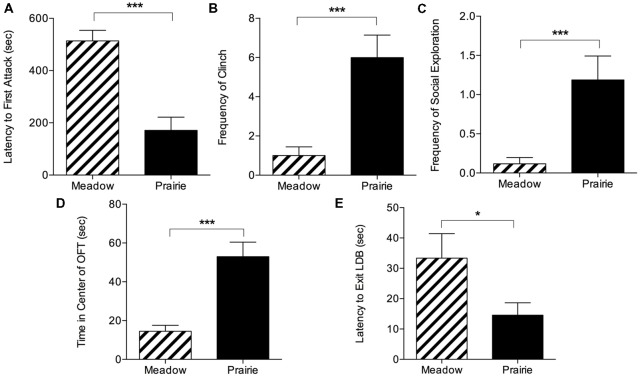
Species differences in behavior during social interaction and anxiety tests. Top panel: in 10-min social interaction tests with a novel conspecific, prairie voles exhibited substantial aggression leading to early test termination. **(A)** Prairie voles showed significantly shorter latency to first attack than meadow voles. Voles who did not attack were given a latency of 600 s. Species comparisons in **(B,C)** were conducted on the maximum interval that included all subjects (~2 min). **(B)** Short day (SD) prairie vole females showed significantly higher frequency of clinch than SD meadow vole females. **(C)** Prairie voles showed significantly higher social exploration than meadow voles. Bottom panel: voles underwent open field tests (OFTs) and light-dark box (LDB) tests. **(D)** OFT: prairie voles spent significantly more time in the center of the open field arena than meadow voles housed in the same day length. **(E)** LDB: prairie voles exited the LDB sooner than meadow voles. *p < 0.05, ***p < 0.0005.

In social interaction tests, SD prairie vole females exhibited aggression sooner and at higher levels than SD meadow vole females. They showed significantly shorter latencies to first (Figure 1A), second, and third attacks compared to SD meadow vole females (*t*_(34)_ = −5.15 for first attack, *t*_(34)_ = −6.72 for second attack, *t*_(34)_ = −7.91 for third attack, *p* < 0.0001 for all). Tests were terminated early significantly more often with prairie voles than meadow voles (12/20 vs. 4/56, *p* < 0.0001, Fisher’s exact test). Because early termination led to uneven test durations, species differences in specific behaviors were assessed using the first 1:51 min of testing, the longest duration that included all tests regardless of whether they were terminated early. Within this time period, SD prairie females exhibited higher frequency of clinch (*t*_(34)_ = 5.04, *p* < 0.0001) and social exploration (*t*_(34)_ = 3.90, *p* < 0.001) compared to SD meadow females (Figures 1B,C).

#### Species Differences in Anxiety-Like Behaviors

Prairie voles exhibited less anxiety-like behavior in the OFT and LDB (Figures 1D,E). In the OFT, prairie voles spent significantly more time in the exposed center than did meadow voles (*t*_(35)_ = 4.86, *p* < 0.0001), and deposited somewhat fewer fecal boli during the test (Mean: 0.89 ± SEM: 0.35 vs. 3.47 ± 1.19), but this difference was not significant (*t*_(35)_ = −2.03, *p* = 0.05). There was no difference in distance traveled between prairie voles (2,181.82 ± 312.60 cm) and meadow voles (1,609.31 ± 130.91 cm; *t*_(35)_ = 1.69, *p* = 0.10). When tested in a LDB, prairie voles were faster to enter the light portion of the box than were meadow voles (*t*_(35)_ = −2.04, *p* = 0.049).

### Study 1b: Effects of Day Length and Social History in Meadow Voles

#### Day Length Effects on Meadow Vole Social and Aggressive Behavior

**Figure 2:**
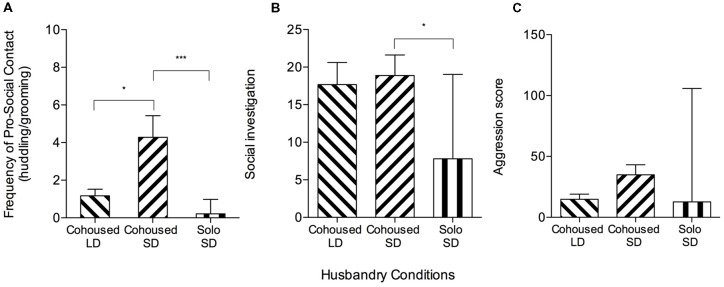
Meadow vole stranger-directed behavior during 10-min social interaction tests. Cohoused SD meadow voles are the same individuals as in Figure 1, but analyzed for full testing intervals to compare to other meadow vole groups. **(A)** There were significant group differences in pro-social contact (one-way ANOVA, *p* < 0.001). Cohoused SD meadow voles showed higher pro-social contact than cohoused long day (LD) meadow voles. Cohoused SD meadow voles showed higher pro-social contact than solo-housed SD meadow voles. **(B)** There were significant group differences in olfactory investigation (sniffing; one-way ANOVA, *p* < 0.05). Cohoused SD meadow voles showed higher sniffing than solo-housed SD meadow voles. **(C)** There were no significant group differences in aggression score across meadow vole groups. *p < 0.05, ***p < 0.0005.

Stranger-directed behaviors differed between SD- and LD-housed meadow voles in the 10-min social interaction test. SD meadow vole females displayed significantly more grooming (*t*_(34)_ = 2.30, *p* = 0.027) and pro-social contact (counts of grooming and huddling; *t*_(34)_ = 2.59, *p* = 0.014, Figure 2A) than did LD meadow vole females. Two conflict-related behaviors were exhibited at higher frequency in SD voles: flight (3.11 ± 0.97 vs. 0.72 ± 0.37, *t*_(34)_ = 2.30, *p* = 0.028) and lateral attack/threat (11.33 ± 2.54 vs. 2.17 ± 2.54, *Z* = 2.19, *p* = 0.02, Wilcoxon rank sum). There were no significant differences in frequency of huddling (*t*_(34)_ = 2.01, *p* = 0.05) or composite aggression score (counts of lateral attack/threat, clinch, upright, and chase; *F*_(2,48)_ = 2.83, *p* = 0.07; Figure 2C).

#### Housing Effects on Meadow Vole Stranger-Directed Interactions

SD meadow females that had been solo-housed from weaning exhibited different social and investigative behaviors than did pair-housed SD meadow females during the social interaction test. Pair-housed meadow vole females displayed significantly more sniffing (*Z* = −2.41, *p* = 0.016, Wilcoxon rank sum), grooming (*t*_(31)_ = −3.32, *p* = 0.002), huddling (*Z* = −3.10, *p* = 0.002), social score (counts of social exploration, sniffing, grooming, and huddling; *t*_(31)_ = −3.51, *p* = 0.001), and pro-social contact (*Z* = −4.23, *p* < 0.0001) than solo-housed meadow vole females (Figures 2A,B). Comparison of solo-housed voles to pair-housed voles also revealed significant differences in frequency of lateral attack/threat (*Z* = −2.11, *p* = 0.035) but not aggression score (*F*_(2,48)_ = 2.83, *p* = 0.07), with pair-housed voles (11.33 ± 2.54) displaying higher frequency of lateral attack/threat than solo-housed voles (3.80 ± 2.78; Figure 2C).

### Study 2: Formation and Reformation of Peer Partner Preferences in Prairie Voles

#### Peer Partner Preferences After Prolonged Cohabitation (PPT 1)

**Figure 3:**
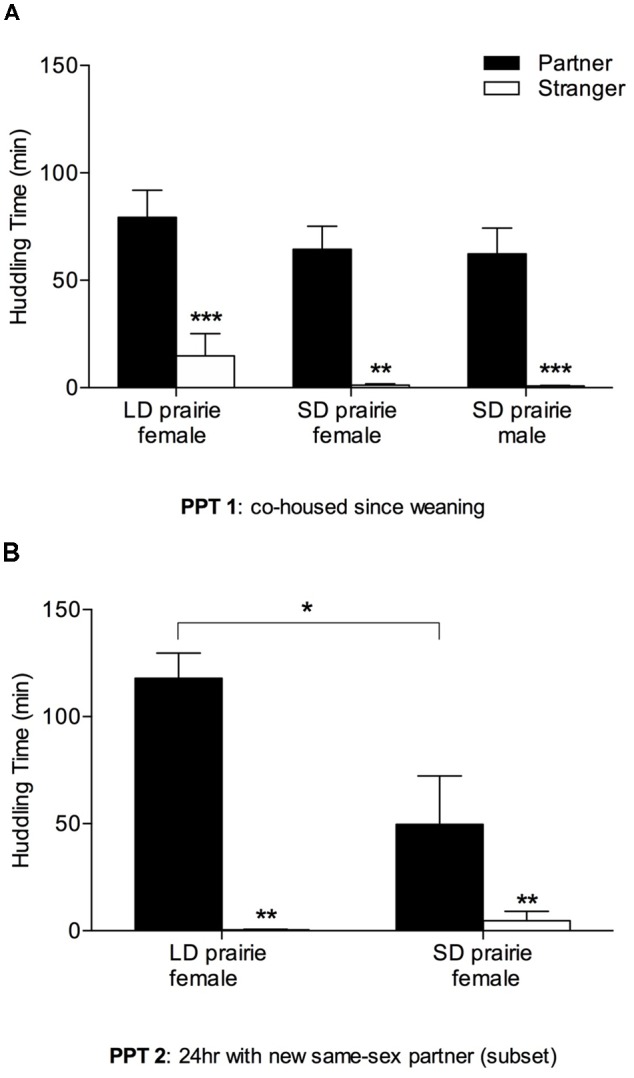
Prairie vole partner preference for stable and new partners. **(A)** Partner preference test 1 (PPT 1; cohoused since weaning): all prairie vole groups (*n* = 11–14) showed robust partner preference for their partners. There were no group differences in partner huddling. **(B)** PPT 2 (24 h with new same-sex partner): a subset of voles was tested for the capacity to form partner preferences for new same-sex partners. Males were not included due to high aggression upon re-pairing. Both female groups (*n* = 8 each) showed robust partner preference for their partners. LD prairie vole females huddled significantly more with their partners than did SD prairie vole females. *p < 0.05, **p < 0.01, ***p < 0.005.

All groups—female prairie voles housed in both day lengths, and males tested in SDs—exhibited partner preferences for same-sex cage-mate partners from weaning, indicated by significantly more huddling with partners than strangers (Figure 3A; LD prairie vole females: *W* = −77.00, *p* = 0.01; SD prairie vole females: *W* = −64.00, *p* = 0.002; SD prairie vole males: *W* = −91.00, *p* < 0.001). No significant differences in partner huddling were evident across groups (*F*_(3,47)_ = 1.86, *p* = 0.15).

#### Peer Partner Preferences and Partner Huddling After Re-pairing (PPT 2)

Both female groups demonstrated the capacity to form preferences for new same-sex partners in adulthood in PPT 2—following 8 days of separation from the first cage-mate and re-pairing with a new cage-mate for 24 h (Figure 3B). Partner huddling was significantly greater than stranger huddling in both LD prairie vole females (*W* = −36.00, *p* = 0.008) and SD prairie vole females (*W* = −64.00, *p* = 0.002). LD prairie vole females huddled significantly more with their partners than did SD prairie vole females (*t*_(14)_ = −2.53, *p* = 0.02). Males were initially tested for the capacity to form new peer partner preferences in adulthood, but the same-sex re-pairing of males was discontinued following the observation of aggression and injuries in the home cage.

## Discussion

These studies are the first to directly compare the peer interactions of meadow and prairie voles, and to consider potential effects of day length on female prairie vole peer affiliation. We also extend findings on anxiety differences between species, and describe how differences in affiliation, aggression, and anxiety may contribute to differences in social structure.

### Prairie Voles Are More Aggressive Than Meadow Voles

Detailed analysis of aggressive and affiliative behavior with novel conspecifics was quantified in social interaction tests. In these tests, SD prairie vole females housed with a peer were highly aggressive toward strangers compared to SD meadow vole females housed with a peer. Social exploration was also higher in prairie voles, usually in advance of the initiation of conflict interactions. This contrasts with a previous finding that meadow voles, not prairie voles, are the more aggressive species (Getz, 1962). However, that study utilized field-caught and laboratory-bred animals which were solo-housed for 2 weeks (field-caught) or 3 months (laboratory-bred) prior to behavioral testing, whereas the longest period of solo-housing in the present study was 1 week (prior to re-pairing with a new same-sex partner in Study 2 to mitigate aggression). It has been well documented that prolonged isolation produces behavioral, physiological, and neuroendocrine changes, at least in prairie voles (Grippo et al., 2008; Lieberwirth et al., 2012).

High aggression toward unfamiliar conspecifics in prairie voles may help to maintain the high selectivity of peer bonds, as it does in pair bonds among mates (Aragona et al., 2006). In other studies of meadow voles, meadow voles have displayed little aggression and high general social contact toward novel same-sex conspecifics (Beery Lab, *unpublished data*). Interaction with strangers in SD meadow voles may be an important avenue for the addition of new members to groups that form in winter.

### Prairie Voles Are Less Anxious Than Meadow Voles

There were robust species differences in anxiety-like behavior in multiple tests. Prior research has shown species differences in anxiety behavior in males (Stowe et al., 2005), which we now extend to females. SD prairie vole females exhibited significantly less anxiety-like behavior than SD meadow vole females in the OFT and the LDB. Prairie voles also exhibited higher levels of social exploration than meadow voles in the social interaction test, consistent with lower anxiety. While used here principally to elicit affiliative and aggressive behaviors, the social interaction test is also a major means of assessing anxiety in rodents (File and Seth, 2003).

Reduced anxiety behavior may be conducive to increased social interaction. In further support of the opposing roles of anxiety and social behavior, exogenous stressors disrupt the formation of partner preferences both in meadow vole females (for peers; Anacker et al., 2016b), and prairie vole females (for mates; DeVries et al., 1996).

### Day Length and Housing Affect Stranger-Directed Behaviors in Meadow Voles

SD meadow vole females displayed significantly higher frequency of social behaviors with novel peers, including grooming and social contact, than LD meadow vole females. This is consistent with higher affiliation toward strangers, expected as SD meadow vole females huddle more with both partners and strangers than LD voles (Beery et al., 2008). Unexpectedly, SD meadow voles also displayed significantly higher frequency of lateral attack/threat behaviors and flight than LD meadow voles. One possible explanation is that SD meadow voles are more willing to engage in interactions of any kind with a conspecific, and that these interactions become more social, or at least more tolerant, over a longer period of time with the conspecific. Social experience may further shape affiliative behaviors, as pair-housed meadow voles showed significantly higher sniffing, grooming, and huddling frequency compared to solo-housed meadow voles. This is consistent with past findings that developmental experiences shape adult social behavior in voles and other species (Bales et al., 2007; Curley et al., 2009; Starr-Phillips and Beery, 2014).

### Prairie Voles Form Peer Partner Preferences Regardless of Day Length or Sex

While social interaction tests assessed stranger-directed behaviors, PPTs assessed affiliation for a familiar animal. All groups cohoused from weaning formed partner preferences for same-sex peers. This places vole social preferences in contrast to those of rats and mice, who do not appear to form selective preferences for familiar peers under ordinary circumstances (Harrison et al., 2016; Schweinfurth et al., 2017; Beery et al., 2018).

Twenty-four hours was sufficient for the formation of new peer partner preferences following separation from old partners in adulthood in females housed in both SD and LD (male groups were discontinued because of aggression). This is consistent with prior findings that LD prairie voles form peer partner preferences within 24 h of cohabitation (DeVries et al., 1997).

Partner huddling was higher in LD prairie vole females than SD prairie vole females in PPT 2. LD prairie vole females displayed partner preferences at consistently high levels in PPT 1 and 2, and re-paired with minimal aggression or need for separation. This supports the use of LD prairie vole females, rather than SD prairie vole females or males, in future studies of peer affiliation in prairie voles. Studying LD prairie vole females for their peer affiliation will also allow for direct comparison with previous work on pair bonding in prairie voles, which was conducted with LD-housed prairie voles.

## Conclusions

Selective partner preferences for same-sex peers appear to be the norm for both prairie and meadow voles, as individuals of each species, sex, and sometimes day length tested here or previously exhibited significant preferences for cage-mates. Social selectivity thus appears to be an important characteristic of social structure in voles. Prairie voles exhibited higher aggression and lower anxiety than meadow voles, and, unlike meadow voles, did not appear to be more affiliative in SD length conditions. In meadow voles, affiliative and aggressive behaviors were altered by day length and by housing. This characterization of peer affiliation, anxiety, and aggression lays the foundations for future work on the mechanisms supporting behavior in different types of peer relationships.

## Data Availability

The datasets generated for this study are available on request to the corresponding authors.

## Author Contributions

All authors designed the study. NL, NG, and KF conducted the research. NL, NG, and AB conducted statistical analyses. NL and AB wrote the manuscript. All authors critically revised the manuscript and gave approval for publication.

## Conflict of Interest Statement

The authors declare that the research was conducted in the absence of any commercial or financial relationships that could be construed as a potential conflict of interest.
